# ToF-SIMS Parallel Imaging MS/MS of Lead Soaps in Embedded Paint Cross Sections

**DOI:** 10.1021/acs.analchem.4c05523

**Published:** 2025-01-06

**Authors:** Kimberly G. Garcia, Philippe Massonnet, Sebastiaan Van Nuffel, Ron M.A. Heeren

**Affiliations:** † Maastricht MultiModal Molecular Imaging (M4i) Institute, Maastricht University, Universiteitssingel 50, Maastricht 6229 ER, The Netherlands

## Abstract

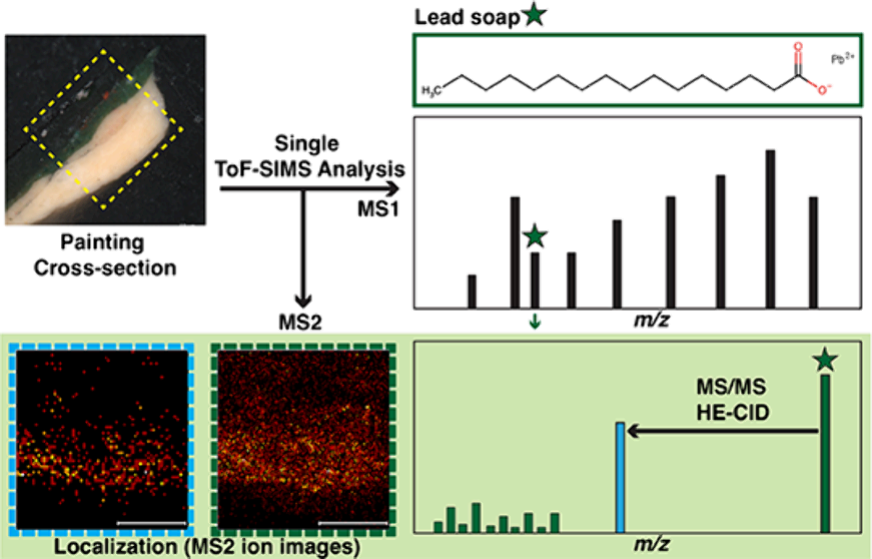

In the field of cultural heritage, and more specifically in oil paintings, the ability to unambiguously identify and locate metal soaps is of great interest for a better understanding of painting degradation. Here, we demonstrate the use of a Time-of-Flight Secondary Ion Mass Spectrometry (ToF-SIMS) instrument capable of tandem mass spectrometry imaging for the unambiguous identification and localization of lead soaps in cross sections of samples of old oil paintings at high spatial resolution. It is shown that the specific fragmentation pattern of lead soaps is dictated by the loss of the lead ion and that fragmentation occurs on the hydrocarbon chains of the fatty acids. This method offers new opportunities for a better understanding of the chemical changes in aging oil paint samples as well as investigation of organic pigments and binders.

## Introduction

Mass spectrometry imaging using Time-of-Flight Secondary Ion Mass Spectrometry or ToF-SIMS offers various advantages in cultural heritage research such as the simultaneous detection of organic and inorganic species with high sensitivity with minimal damage at a high spatial resolution.^1−12^ In ToF-SIMS, a primary ion beam is pulsed toward a surface under static conditions (<10^13^ ions per cm^2^) causing the desorption and ionization of molecules from the very top of the sample’s surface. Therefore, ToF-SIMS is a relatively nondestructive analytical technique, which is an important consideration when analyzing precious cultural heritage samples.

Oil paintings are made by successive layers of pigments and one or multiple binders such as oil.^13,14^ Metal soap formation is most often associated with oil painting degradation and a good understanding of how these species are formed is therefore important.^1,15,16^ Metal soaps are a combination of heavy metals or alkaline earth elements, originating from the inorganic pigments, with carboxylic acids, originating from the oil-binding medium.^17^ Metal soap formation in oil paintings has been studied using different methods such as optical microscopy, differential scanning calorimetry (DSC), Energy Dispersive X-ray spectroscopy (SEM-EDX), and various spectroscopy techniques such as attenuated total reflection Fourier transform infrared spectroscopy (ATR-FTIR), infrared (IR), and Raman spectroscopy.^1,17−19^ However, these techniques cannot definitively chemically identify metal soaps.

ToF-SIMS analysis of metal-soap species such as lead palmitate and lead stearate has also been performed.^3,4,8,11,12,20^ In imaging mode, these lead soaps can be localized with high spatial resolution (<1 μm). In these studies, metal-soap identities have been assessed based on *m*/*z* value and isotopic pattern matching. Although these robust indicators allow for a tentative assignment, the identities of these lead soaps have not been confirmed unambiguously using tandem mass spectrometry. This has complicated the full characterization of such complexes. New instrumental developments in TOF-SIMS imaging have made it possible to obtain MS/MS data in parallel with ToF-SIMS imaging.^21^ For MS1 imaging, an MS1 spectrum is recorded for every pixel; thus, an ion image from each ion peak in the spectra can be created. During parallel imaging, MS/MS or MS2, MS1 ion images, and an MS2 ion image data set are simultaneously created with the same raster and pixel image density. Ion images from the selected fragment ions can also be created, as each pixel in the image has a full MS2 spectrum. However, a much longer MS2 acquisition is needed to accumulate the ion counts to make an image comparable to the MS1 image due to the lower abundance of ions collected for each frame during MS2 isolation.

The instrument is also equipped with a dual-beam charge neutralization system for charge compensation of challenging samples such as insulators.^22,23^ Charge compensation is necessary in the case of insulating samples to offset the buildup of positive charge due to the constant bombardment of the positive ion beam.^24,25^ The electron gun neutralizer (E-neut) works by aiming a low energy electron (<25 eV) beam toward the sample to mitigate the accumulation of positive charge build-up in either polarity.^24^ The ion neutralizer (I-neut) is used to eliminate static charge present on insulation samples charge buildup during the analysis of a flood of Ar^+^ ions (<10 eV) during positive mode.^22,24^ As painting samples are typically embedded in thick resin blocks, which are insulators, the use of these technologies is necessary.

In this study, lead soap compounds in a painting cross-section are characterized using a TOF-SIMS instrument with parallel MS/MS imaging capabilities and a dual-beam charge compensation system to evaluate the benefit of the combined use of ToF-SIMS and tandem mass spectrometry in the field of cultural heritage. The results presented in this study open the path for a workflow for the full characterization of metal soaps in paint cross-section samples.

## Materials and Methods

### Samples

Cross-section extracts from an old painting were first embedded in Polypol polyester resin and then hand polished until the different layers became accessible for ion beam sampling. A diamond knife was used to cut an elevated platform for the embedded painting sample.

### TOF-SIMS Analysis

TOF-SIMS analyses were performed on a PHI nanoTOF II instrument (Physical Electronics, Chanhassen, MN, USA) equipped with a 30 keV Bi_n_^q+^ liquid metal ion gun (LMIG) used as an analysis beam and an Ar gas cluster ion beam (GCIB; 10 keV) for nondestructive sputter cleaning. Charge compensation was done using the dual charge neutralization system and adjusting sample bias to maintain a stable ion count (see Supplementary Figure S1). In either polarity, an electron gun neutralizer (25 eV electron beam) was used, while in the positive mode, an ion neutralizer (10 eV Ar^+^ ions) was used additionally. The sample bias was increased by +405 V compared to the normal sample bias value. Before each measurement, 30 s of Ar GCIB sputtering (DC: 10 nA, 1000 μm × 1000 μm raster size) was performed to remove contaminants on the surface (See Supplementary Figure S2). Negative and positive polarity TOF-SIMS images were acquired on the same location using Bi_3_^+^ as the primary ion beam, with a 200 μm aperture resulting to a DC of 3.0 nA, using a mass range from 0 to 1850 *m*/*z* over one area of 256 μm × 256 μm with 256 × 256 pixels for both MS1 and MS2. MS1 only imaging was done over 50 frames resulting in primary ion doses of 6.18 × 10^10^ ions/cm^2^. Parallel MS1 and MS2 imaging was done over a longer acquisition of 499 frames to accumulate enough signals for the MS2 detector. The primary ion dose for parallel imaging was calculated to be 6.17 × 10^11^ ions/cm^2^.

Negative ion spectra were calibrated using O_2_^–^ at *m*/*z* 31.9898, PbO_2_H^–^ at *m*/*z* 240.9742, ^206^PbPbO_3_H^–^ at *m*/*z* 462.9436, ^206^PbPb_2_O_4_H^–^ at *m*/*z* 686.9151. Positive ion spectra were calibrated using Na^+^ at *m*/*z* 22.9898, Pb^+^ at *m*/*z* 207.9766, Pb_2_O^+^ at *m*/*z* 431.9481, ^206^PbPb_2_O_2_^+^ at *m*/*z* 653.9175, and ^206^PbPb_3_O_3_^+^ at *m*/*z* 877.8890. Tandem MS was performed in positive polarity. The MS/MS settings used in this study were previously described with a precursor selector window of 1 Da.^26^

### Data Analysis

All data was processed using the commercial PHI software (TOF-DR 3.3.0.19). Pigment identifications were done through pigment literature comparison, isotope matching, and mass accuracy (<20 ppm).

### Optical Microscopy

Bright-field and dark-field microscopy images were acquired using a Leica DMRX microscope (Leica Biosystems, Amsterdam, The Netherlands) equipped with a Nikon DXM1200 digital camera. A 10x objective lens was used. All images were processed by using Leica LAS 3.8 software.

## Results and Discussion

**Figure 1:**
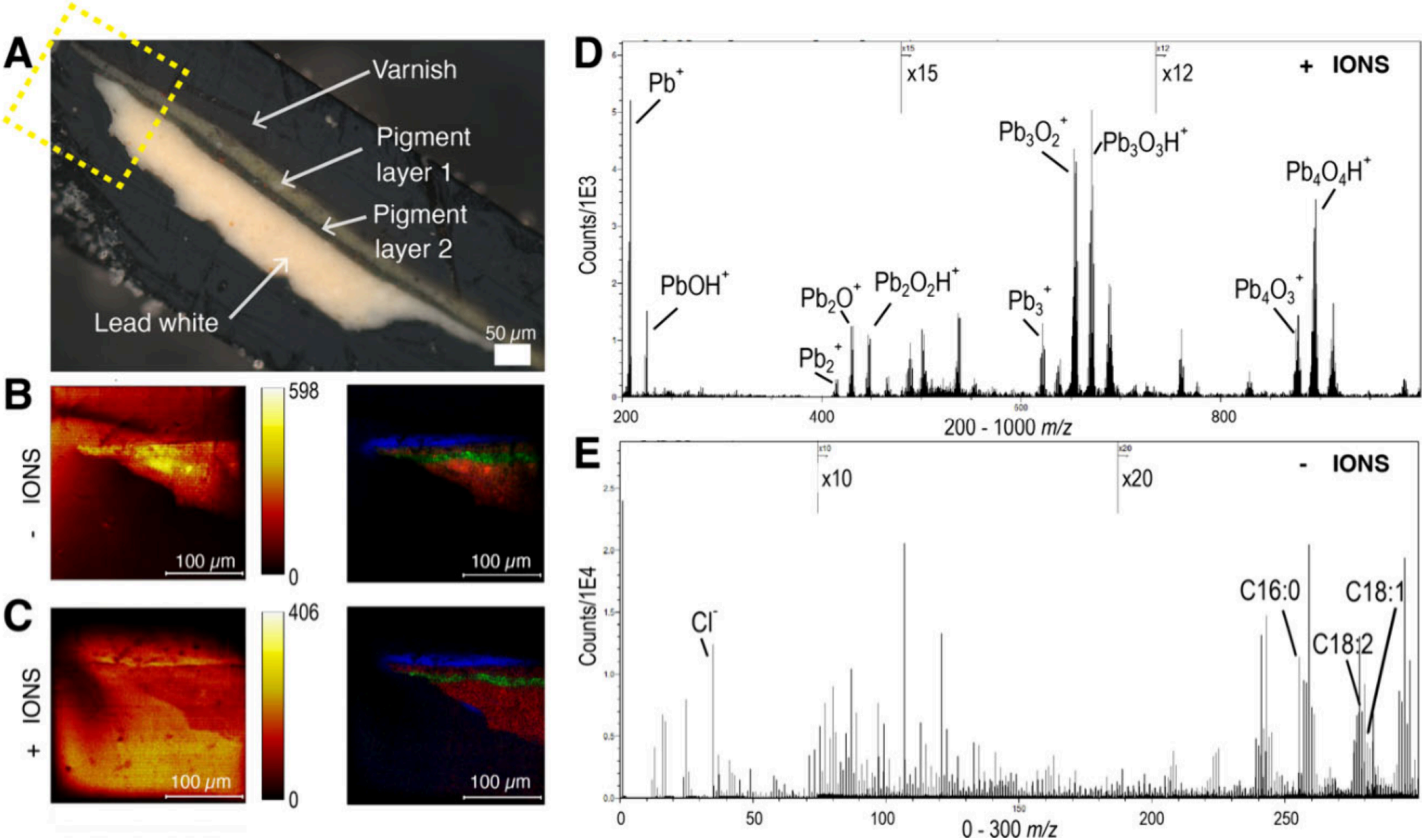
Sample 1 shows the different layers that are present. A) The optical image of the paint sample with 4 different layers labeled, and the analysis area in the box. B) ToF-SIMS ion images in the negative ion mode showing (right) total ion image and (left) overlaid ion images of [C_2_H_3_O_2_]^−^ at 59.01 *m*/*z* (blue), [PbO_2_H]^−^ at 240.97 *m*/*z* (red), and *unknown* at 105.94 *m*/*z* (green) showing the different layers of the painting. C) ToF-SIMS ion images in the positive ion mode showing (right) total ion image and (left) overlaid ion images of [C_2_H_3_O]^+^ at 43.02 *m*/*z* (blue), Pb^+^ at 207.98 *m*/*z* (red), and Cu^+^ at 62.93 *m*/*z* (green) showing the same different layers. D) Positive ion spectra from ROI of the lead white layer from 200–1000 *m*/*z*, showing ion signals consistent with lead white pigment compounds such as lead oxides and lead carboxylates. E) Negative ion spectra from ROI of the lead white layer from 0–300 *m*/*z*, showing ion signals for fatty acids such as palmitic acid (C16:0), oleic acid (C18:1), and linoleic acid (C18:2).

Three painting samples were analyzed, which are part of the same oil painting. The dual beam charge compensation and bias adjustment have resulted in a stable ion count over the whole analysis to achieve the following ion images (See Supplementary Figure S1). The optical image of sample 1 is shown in Figure 1A, with the area of analysis marked. Positive and negative ion ToF-SIMS showed unique chemical information for each layer for sample 1.

Spectra in both positive and negative modes were extracted from regions of interest (ROI) for each layer showing the different unique chemical information and pigments used (Figure 1B and 1C; see Supplementary Figures S4, S5). The lateral resolution (Δ*l*_80/20_) was measured using a curve fit to line scans of 5-pixel average line width and using the 80% and 20% bounds of the curve (Supplementary Figure S3). Line scans using the 3 different layers for each polarity shown (Figure 1B, 1C) resulted in average Δ*l*_80/20_ of 1.86 and 1.98 μm for positive mode and negative mode, respectively.

The varnish layer contains organic signals such as [C_6_H_5_]^+^, [C_7_H_7_]^+^, and [C_2_H]^−^, with the dominant peak of [CH_3_COO]^−^ and [C2H3O]^+^ (Supplementary Figure S4, S5). Pigment layer 2 is distinguished by strong ion signals for Cu^+^ and [Cu_3_O]^+^, indicating the presence of a green to blue-green pigment such as malachite or verdigris.^27^ Pigment layer 1 is distinguished by barium-containing ion signals ([Ba_2_O_2_H]^+^ and [BaOH]^+^) in positive polarity and by sulfur-containing ion signals ([SO_2_]^−^ and [SO_3_]^−^) in negative polarity, which is characteristic of barium sulfate, a white pigment often used as a filler. Pigment layer 1 is also shown to contain numerous characteristic ions from lead white as well as minor ion signals for Cu^+^ and [Cu_3_O]^+^, which suggests that the layer consists of a mixture of lead white, barium sulfate, copper pigment, and perhaps others to form a lighter green/blue-green hue.

The white layer of the painting contains lead white pigment compounds such as lead oxides, and lead carboxylates (Figure 1D, Supplementary Figures S4, S5).^28,29^ In the same layer, fatty acids are found, for example, palmitic acid (C16:0), stearic acid (C18:0), oleic acid (C18:1), and linoleic acid (C18:2), among others. These fatty acids are proposed to be reactive with metals such as lead to produce lead soaps, which build up over time and form protrusions that may cause damage to a painting.^15,20^ In this study, stearic acid lead soap [C_18_H_35_O_2_^–^Pb^2+^]^+^ at 491.23 *m*/*z* and palmitic acid lead soap [C_16_H_31_O_2_^–^Pb^2+^]^+^ at *m*/*z* 463.21 were detected as small peaks in the positive ion mode.

**Figure 2:**
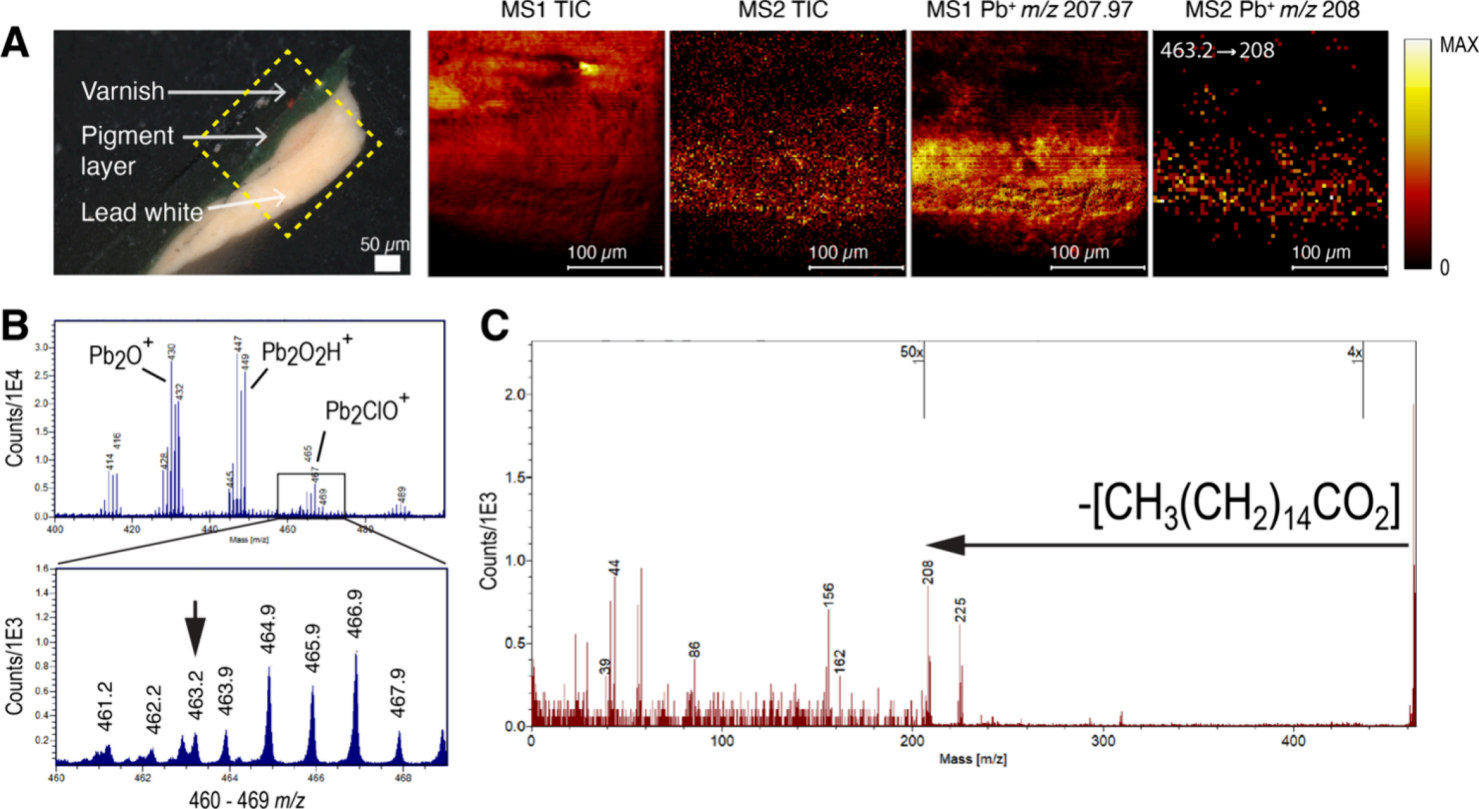
Parallel MS/MS imaging of palmitic acid lead soap from sample 2. A) Optical image of the sample and analysis area marked by a box. The ion images for MS1 (TIC and Pb^+^ at *m*/*z* 208.0) and MS2 (TIC and Pb^+^ at *m*/*z* 208.0) are presented (scale bars at 100 μm). B) Mass spectra without tandem MS showing the palmitic acid lead soap peak at *m*/*z* 463.2. C) MS2 fragmentation of the palmitic acid lead soap peak at *m*/*z* 463.2 isolated using the tandem MS 1 Da window.

Tandem MS imaging for the palmitic acid lead soap peak (Figure 2B) was performed to verify and characterize the palmitic acid lead soap in different paint samples. Despite the low abundance of this peak, it is possible to isolate such low abundance compounds using a 1 Da isolation window and with a long acquisition using parallel MS/MS imaging.^30,31^ Sample 2 is shown below with its optical image shown in Figure 2A. The MS1 total ion image (TIC) and the MS2 TIC from the isolated palmitic acid lead soap peak at *m*/*z* 463.21 are shown.

The MS2 fragmentation data show the full fatty acid loss, with the highest fragment ion being Pb^+^ (Figure 2C). This is consistent with the instruments’ high energy collision-induced dissociation (HE-CID, 1.5 keV) process where the weakest bonds are broken first.^26^ Additionally, an MS2 [PbOH]^+^ peak was also found at 224.98 *m*/*z* indicating a strong interaction with the lead and oxygen from the fatty acid chain. Lower mass fragments show fragmentation of the aliphatic chain group. The same observations are found for sample 3 (Supplementary Figure S6).

In Figure 2A, the differences between the MS1 ion image of Pb^+^ at *m*/*z* 207.97 from the MS2 ion image of Pb^+^ are shown. The MS2 Pb^+^ fragment ion image originates solely from the lead soap and shows it is localized mostly in the lead white layer; however, some of it can already be observed in the other layers. This suggests the formation of the lead soap in the other pigment layer.

## Conclusion

The goal of this study was to evaluate the potential and use of ToF-SIMS parallel MS/MS imaging to investigate lead soap species found in embedded paint cross sections.

Using this instrumental setup, tandem mass spectrometry of palmitic acid lead soaps of embedded paint cross sections was performed for the first time. The advantage of this setup is that it is able to get localization information while doing MS/MS, making the structural characterization of lead soaps possible during imaging at high lateral resolution. Spatial localization can indeed be used as a filter to only obtain relevant chemical information, providing ultimate proof of identification. The obtained MS2 spectra show the full loss of fatty acids as well as fragmentation around the Pb–O coordination site(s). These results open the possibility of investigating metal–oxygen coordination sites using tandem mass spectrometry. This approach can be applied to other metal soaps such as zinc lead soaps and makes it possible to investigate the metal-soap formation in paintings by sampling metal soaps at different time points or using artificial painting aging.

## Supporting Information Available

Figure S1: Comparison of TIC profile for the sample with charge compensation off and without charge compensation on. Figure S2: Removal of surface contaminants using Ar GCIB. Figure S3: Calculation of lateral resolution using different ion images. Figure S4: Positive ion spectra extracted from each layer of sample 1. Figure S5: Negative ion spectra extracted from each layer of sample 1. Figure S6: Parallel MS/MS Imaging of palmitic acid lead soap of sample 3. The raw ToF-SIMS data sets can be downloaded directly from DataverseNL at 10.34894/LRIH3A (PDF)

## ABBREVIATIONS

ToF-SIMS: Time-of-flight Secondary Ion Mass Spectrometry
ROI: region of interest
TIC: total ion image
HE-CID: high energy collision-induced dissociation
MS: mass spectrometry
GCIB: gas cluster ion beam
LMIG: liquid metal ion gun

## References

[ref1] Izzo F. C.; Kratter M.; Nevin A.; Zendri E. A Critical Review on the Analysis of Metal Soaps in Oil Paintings. ChemistryOpen 2021, 10, 904–921. 10.1002/open.202100166. 34532965 PMC8446710

[ref2] Bouvier C.; Van Nuffel S.; Walter P.; Brunelle A. Time-of-flight Secondary Ion Mass Spectrometry Imaging in Cultural Heritage: A Focus on Old Paintings. J. Mass Spectrom. 2022, 57, e4803 10.1002/jms.4803. 34997666

[ref3] Iorio M.; Sodo A.; Graziani V.; Branchini P.; Casanova Municchia A.; Ricci M. A.; Salvadori O.; Fiorin E.; Tortora L. Mapping at the Nanometer Scale the Effects of Sea-Salt Derived Chlorine on Cinnabar and Lead White by Using Delayed Image Extraction in ToF-SIMS. Analyst 2021, 146, 2392–2399. 10.1039/D0AN02350G. 33656508

[ref4] Atrei A.; Scala A.; Giamello M.; Uva M.; Pulselli R. M.; Marchettini N. Chemical Composition and Micro Morphology of Golden Laminae in the Wall Painting “La Maestà” by Simone Martini: A Study by Optical Microscopy, XRD, FESEM-EDS and ToF-SIMS. Appl. Sci. 2019, 9, 3452 10.3390/app9173452.

[ref5] Bouvier C.; Glanville H.; de Viguerie L.; Merucci C.; Walter P.; Brunelle A. Time-of-Flight Secondary Ion Mass Spectrometry Imaging of Cross Sections from the Bacchanals Paintings of Nicolas Poussin. Anal. Chem. 2021, 93, 4463–4471. 10.1021/acs.analchem.0c04471. 33661602

[ref6] Biocca P.; Santopadre P.; Sidoti G.; Sotgiu G.; de Notaristefani F.; Tortora L. ToF-SIMS Study of Gilding Technique in the Fresco Vela Della Castità by Giotto’s School. Surf. Interface Anal. 2016, 48, 404–408. 10.1002/sia.5956.

[ref7] Ingo G. M.; Riccucci C.; Pascucci M.; Messina E.; Giuliani C.; Biocca P.; Tortora L.; Fierro G.; Di Carlo G. Combined Use of FE-SEM+EDS, ToF-SIMS, XPS, XRD and OM for the Study of Ancient Gilded Artefacts. Appl. Surf. Sci. 2018, 446, 168–176. 10.1016/j.apsusc.2018.01.278.

[ref8] Sodo A.; Tortora L.; Biocca P.; Casanova Municchia A.; Fiorin E.; Ricci M. A. Raman and Time of Flight Secondary Ion Mass Spectrometry Investigation Answers Specific Conservation Questions on Bosch Painting Saint Wilgefortis Triptych. J. Raman Spectrosc. 2019, 50, 150–160. 10.1002/jrs.5479.

[ref9] de Ghetaldi K.; Wiggins M. B.; Bertorello C.; Voras Z.; Norbutus A.; Beebe T. P. Jr; Baade B. In-Depth Examination and Analysis of Domenico Cresti’s Oil on Wall Paintings in Santa Maria Della Pace in Rome. J.Cult. Herit. 2017, 28, 48–55. 10.1016/j.culher.2017.05.001.

[ref10] Voras Z. E.; de Ghetaldi K.; Wiggins M. B.; Buckley B.; Baade B.; Mass J. L.; Beebe T. P. ToF-SIMS Imaging of Molecular-Level Alteration Mechanisms in Le Bonheur de Vivre by Henri Matisse. Appl. Phys. A:Mater. Sci. Process. 2015, 121, 1015–1030. 10.1007/s00339-015-9508-2. 27482144 PMC4959045

[ref11] Tortora L.; de Notaristefani F.; Ioele M. ToF-SIMS Investigation of Gilt and Painted Leather: Identification of Indigo, Oil Binder and Gold Varnish. Surf. Interface Anal. 2014, 46, 807–811. 10.1002/sia.5450.

[ref12] Sanyova J.; Cersoy S.; Richardin P.; Laprévote O.; Walter P.; Brunelle A. Unexpected Materials in a Rembrandt Painting Characterized by High Spatial Resolution Cluster-TOF-SIMS Imaging. Anal. Chem. 2011, 83, 753–760. 10.1021/ac1017748. 21218778

[ref13] Baij L.; Chassouant L.; Hermans J. J.; Keune K.; Iedema P. D. The Concentration and Origins of Carboxylic Acid Groups in Oil Paint. RSC Adv. 2019, 9, 35559–35564. 10.1039/C9RA06776K. 35528099 PMC9074637

[ref14] Bouvier C.; Brunelle A.; Van Nuffel S. Transferable Mass Spectrometry Methods: Examination of Authenticity in Artwork. Applications of Mass Spectrometry for the Provision of Forensic Intelligence 2023, 236–264.

[ref15] Eumelen G. J. A. M.; Bosco E.; Suiker A. S. J.; Hermans J. J.; van Loon A.; Keune K.; Iedema P. D. Computational Modelling of Metal Soap Formation in Historical Oil Paintings: The Influence of Fatty Acid Concentration and Nucleus Geometry on the Induced Chemo-Mechanical Damage. SN Appl. Sci. 2020, 2, 1310 10.1007/s42452-020-3038-z.

[ref16] Cotte M.; Checroun E.; Susini J.; Walter P. Micro-Analytical Study of Interactions between Oil and Lead Compounds in Paintings. Appl. Phys. A:Mater. Sci. Process. 2007, 89, 841–848. 10.1007/s00339-007-4213-4.

[ref17] Robinet L.; Corbeil-a M.-C. The Characterization of Metal Soaps. Stud. Conserv.Stud. Conserv. 2003, 48, 23–40. 10.1179/sic.2003.48.1.23.

[ref18] Cotte M.; Checroun E.; De Nolf W.; Taniguchi Y.; De Viguerie L.; Burghammer M.; Walter P.; Rivard C.; Salomé M.; Janssens K.; Susini J. Lead Soaps in Paintings: Friends or Foes?. Stud. Conserv. Stud. Conserv. 2017, 62, 2–23. 10.1080/00393630.2016.1232529.

[ref19] Raven L. E.; Bisschoff M.; Leeuwestein M.; Geldof M.; Hermans J. J.; Stols-Witlox M.; Keune K. Delamination Due to Zinc Soap Formation in an Oil Painting by Piet Mondrian (1872–1944). Cultural Heritage Science 2019, 343–358. 10.1007/978-3-319-90617-1_20.

[ref20] Keune K.; Boon J. J. Analytical Imaging Studies of Cross-Sections of Paintings Affected by Lead Soap Aggregate Formation. Stud. Conserv. 2007, 52, 161–176. 10.1179/sic.2007.52.3.161.

[ref21] Fisher G. L.; Hammond J. S.; Larson P. E.; Bryan S. R.; Heeren R. M. A. Parallel Imaging MS/MS TOF-SIMS Instrument. J. Vac. Sci. Technol., B:Nanotechnol. Microelectron.:Mater., Process., Meas., Phenom. 2016, 34, 3–126. 10.1116/1.4943568.

[ref22] Baer D. R.; Artyushkova K.; Cohen H.; Easton C. D.; Engelhard M.; Gengenbach T. R.; Greczynski G.; Mack P.; Morgan D. J.; Roberts A. XPS Guide: Charge Neutralization and Binding Energy Referencing for Insulating Samples. J. Vac. Sci. Technol., A 2020, 38, 31204 10.1116/6.0000057.

[ref23] Edwards L.; Mack P.; Morgan D. J. Recent Advances in Dual Mode Charge Compensation for XPS Analysis. Surf. Interface Anal. 2019, 51, 925–933. 10.1002/sia.6680.

[ref24] Physical Electronics USA; ULVAC-PHI INC. TRIFT III TOF SIMS Operator’s Guide; PN649277 Rev C.; ULVAC-PHI Inc.: Chigasaki, JAPAN.

[ref25] Hunt C. P.; Stoddart C. T. H.; Seah M. P. The Surface Analysis of Insulators by SIMS: Charge Neutralization and Stabilization of the Surface Potential. Surf. Interface Anal. 1981, 3, 157–160. 10.1002/sia.740030404.

[ref26] Bryan S. R.; Fisher G. L.; Larson P. E.; Hammond J. S.; Heeren R. M. A.; Iida S.; Miyayama T. Use of High Energy Collision Induced Dissociation (HE-CID) in TOF-SIMS for Unambiguous Peak Identification and Imaging. J. Surf. Anal. 2017, 24, 167–169. 10.1384/jsa.24.167.

[ref27] Bouvier C.; Van Nuffel S.; Brunelle A. Tof-SIMS Spectra of Historical Inorganic Pigments: Copper-, Zinc-, Arsenic-, and Phosphorus-Containing Pigments in Positive Polarity. Surf. Sci. Spectra 2024, 31, 025001 10.1116/6.0003722.

[ref28] Bouvier C.; Van Nuffel S.; Brunelle A. ToF-SIMS Spectra of Historical Inorganic Pigments: Lead-Based Pigments in Positive Polarity. Surf. Sci. Spectra 2024, 31, 015003 10.1116/6.0003507.

[ref29] Bouvier C.; Van Nuffel S.; Brunelle A. ToF-SIMS Spectra of Historical Inorganic Pigments: Lead-Based Pigments in Negative Polarity. Surf. Sci. Spectra 2024, 31, 015005 10.1116/6.0003560.

[ref30] Bruinen A. L.; Fisher G. L.; Balez R.; van der Sar A. M.; Ooi L.; Heeren R. M. A. Identification and High-Resolution Imaging of α-Tocopherol from Human Cells to Whole Animals by TOF-SIMS Tandem Mass Spectrometry. J. Am. Soc. Mass. Spectrom. 2018, 29, 1571–1581. 10.1007/s13361-018-1979-x. 29949055 PMC6060986

[ref31] Fu T.; Touboul D.; Della-Negra S.; Houël E.; Amusant N.; Duplais C.; Fisher G. L.; Brunelle A. Tandem Mass Spectrometry Imaging and in Situ Characterization of Bioactive Wood Metabolites in Amazonian Tree Species *Sextonia Rubra*. Anal. Chem. 2018, 90, 7535–7543. 10.1021/acs.analchem.8b01157. 29856602

