# Supplementary material for: ToF-SIMS Parallel Imaging MS/MS of Lead Soaps in Embedded Paint Cross Sections
Source: Anal Chem. 2025 Jan 6;97(2):1054–8. doi: 10.1021/acs.analchem.4c05523 (PMC11755392; doi:10.1021/acs.analchem.4c05523)
Supplement: Supplementary file 1 — ac4c05523_si_001.pdf [file ac4c05523_si_001.pdf]

Supporting Information

ToF-SIMS Parallel Imaging MS/MS of Lead Soaps in Embedded Paint Cross Sections

Kimberly G. Garcia[1], Philippe Massonnet[1], Sebastiaan Van Nuffel[1], Ron M.A. Heeren[1]*

[1] Maastricht MultiModal Molecular Imaging (M4i) Institute, Maastricht University, Universiteitssingel 50, 6229 ER Maastricht, The Netherlands

Table of Contents

Figure S1 .... S2
Figure S2 .... S2
Figure S3 .... S3
Figure S4 .... S4
Figure S5 .... S5
Figure S6 .... S6

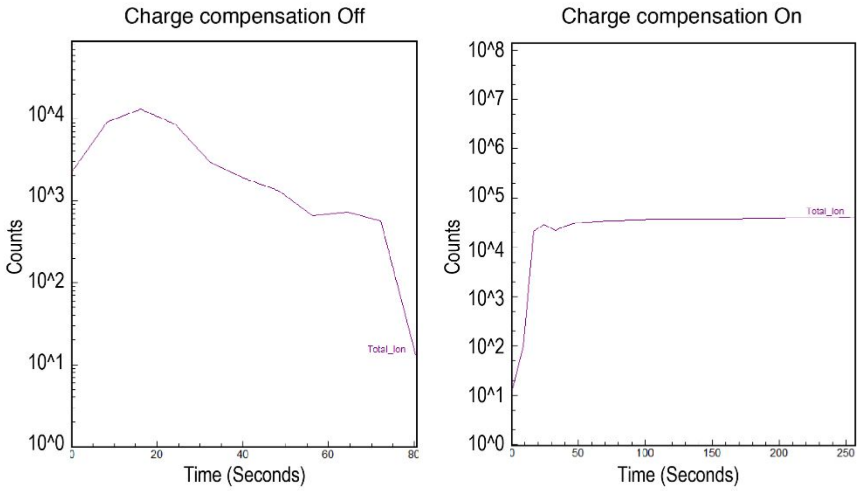

Figure S1. Profile of the Total Ion Count (TIC) during analysis in the positive mode at the same increased sample bias of +3505 V. (Left) Dual charge compensation is off. TIC slowly decreases until 0 ion counts after 80 seconds. (Right) Dual charge compensation, E-neut, and I-neut activated. TIC is in plateau even after 80 seconds and is maintained throughout the experiment. The increase in signal after 1-2 seconds is always observed with dual charge compensation on.

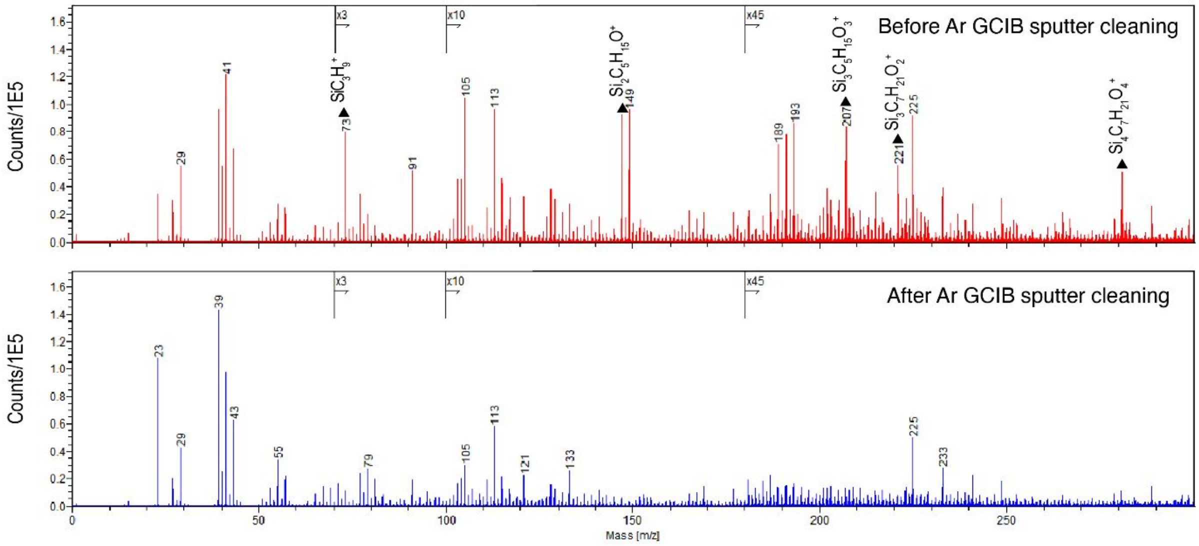

Figure S2. Removal of surface contaminants using sputter cleaning with Ar cluster GCIB. Here is the mass spectrum of a 256 x 256 µm area of the resin block adjacent to the embedded painting. (Top) The spectra before GCIB sputter cleaning. Marked are 5 peaks associated with a common surface contaminant polydimethylsiloxane (PDMS). (Bottom) Mass spectra of the same area after 30 seconds GCIB sputter cleaning showing the PDMS peaks are no longer dominant in the spectra after Ar sputter cleaning. Other peaks, presumably volatile organics, are also removed.

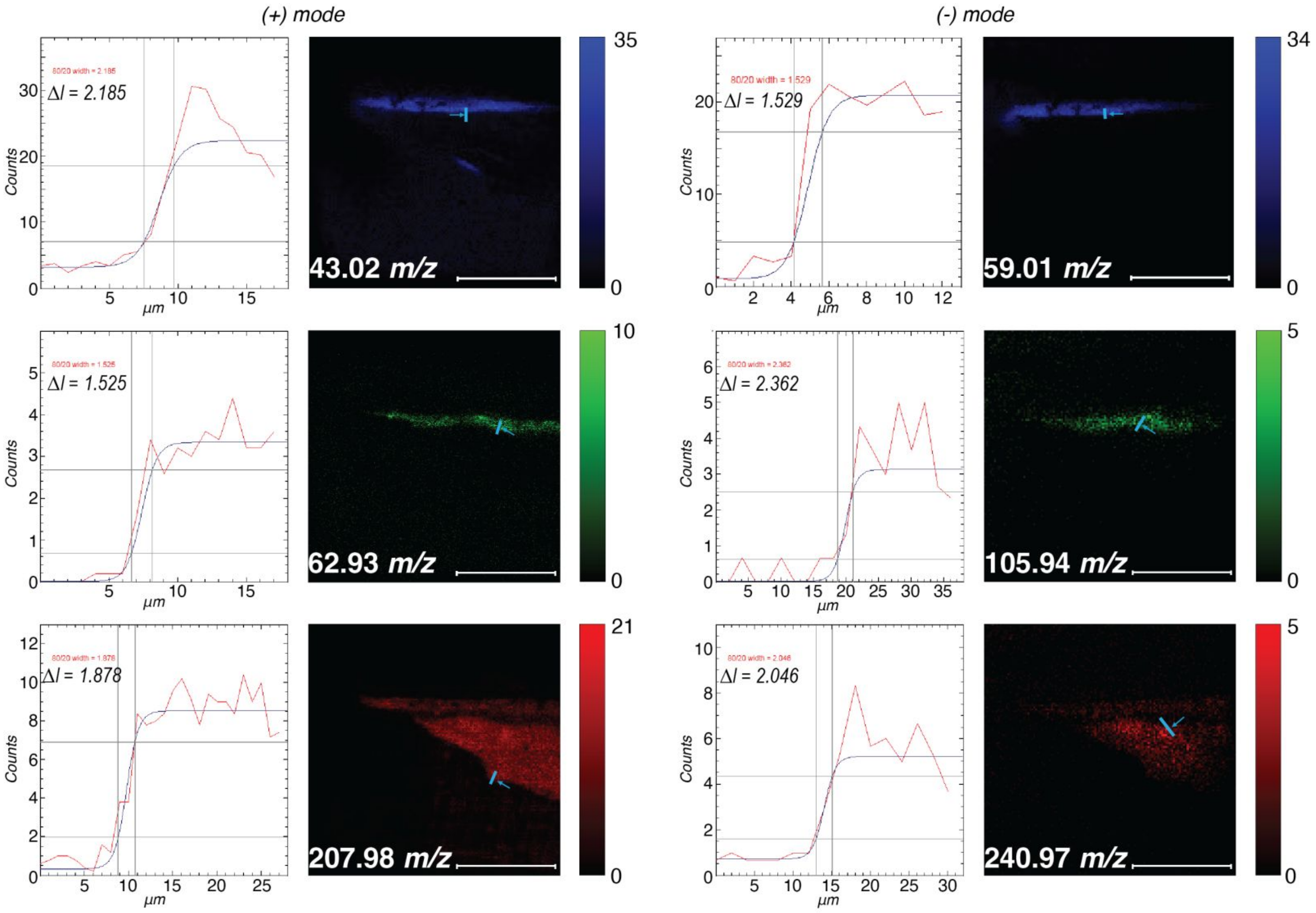

Figure S3. Lateral resolution ($\Delta l_{80/20}$) measurement of the representative ion images showing different layers in both polarities. Marked areas (blue lines and arrow) indicate where the line scans (average line width of 5 pixels) were done. Calculations of the 80% and 20% bounds of the curve fit to the line scans were done using the commercial PHI software (ToF-DR 3.3.0.19). Scale bars at 100 µm.

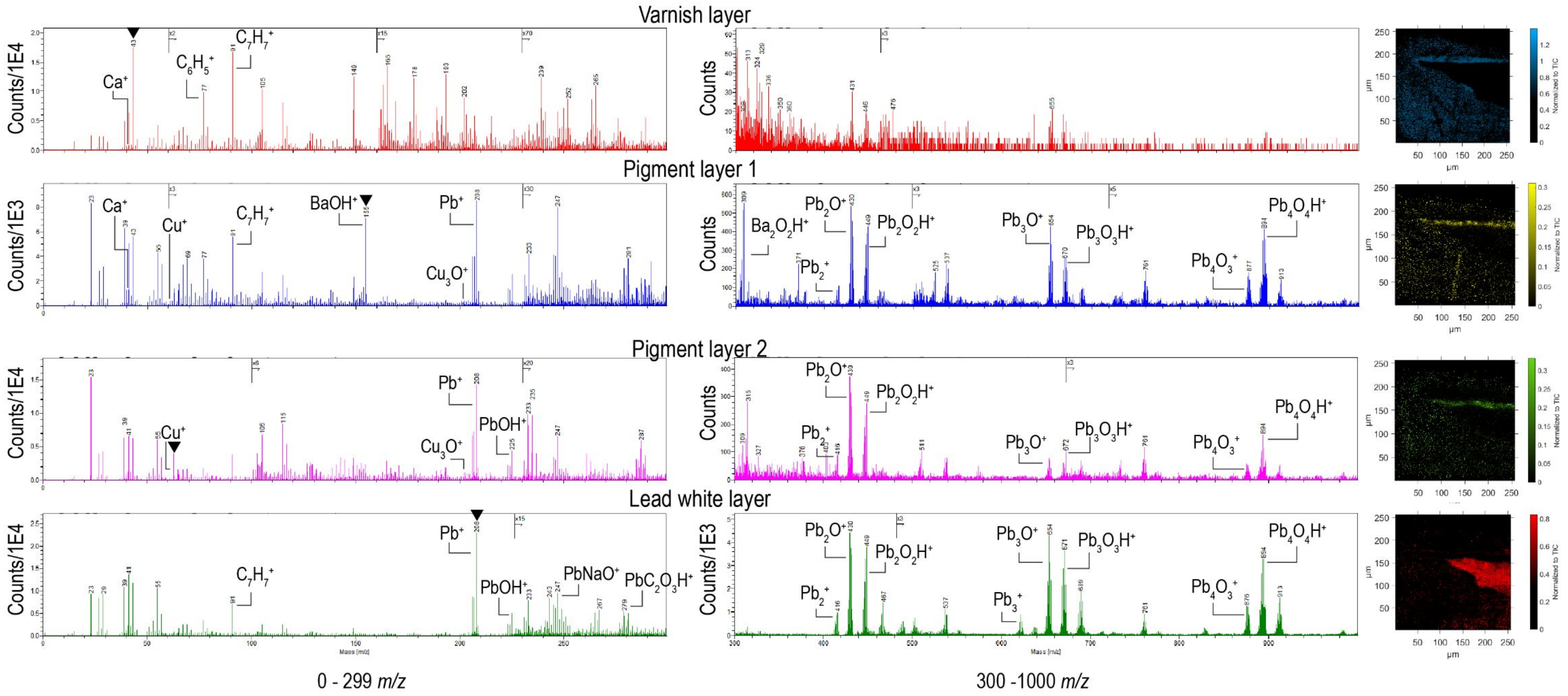

Figure S4. Positive ion spectra extracted from regions of interest (ROI) from each identified layer in the painting sample. Representative normalized ion images from each layer are also shown (marked with inverted triangles). ROIs were obtained using thresholding using a representative high abundance ion for each layer, specifically, (ROI 1, 1st row) $[C_2H_3O]^+$ at 43.02 *m/z*, (ROI 2, 2nd row) $[BaOH]^+$ at 154.90 *m/z*, (ROI 3, 3rd row) Cu^+ at 62.93 *m/z*, and (ROI 4, 4th row) Pb^+ at 207.98 *m/z*.

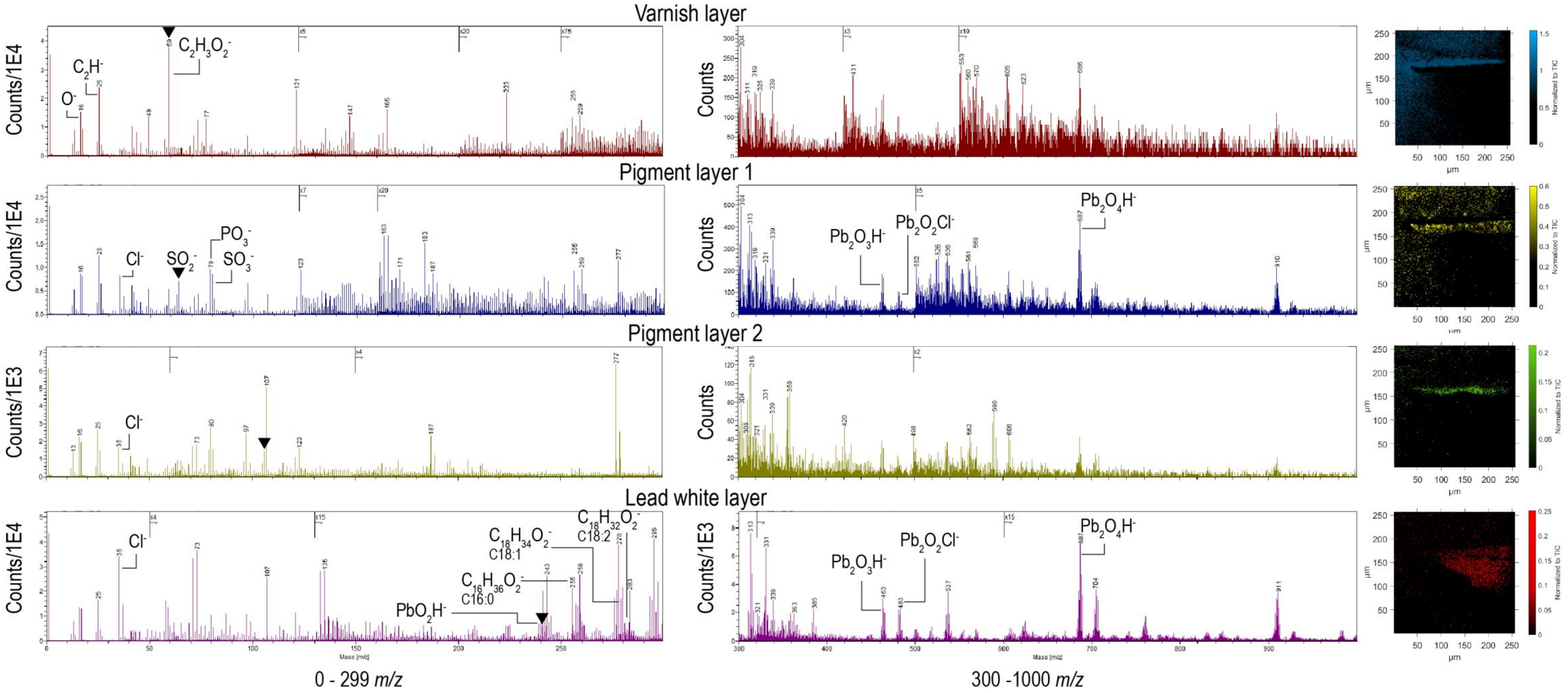

Figure S5. Negative ion spectra extracted from regions of interest (ROI) from each identified layer in the painting sample. Representative normalized ion images from each layer are also shown (marked with inverted triangles). ROIs were obtained using thresholding representative high abundance ion for each layer, specifically, (ROI 1, 1st row) $[C_2H_3O_2]^-$ at 59.01 *m/z*, (ROI 2, 2nd row) $[SO_2]^-$ at 63.96 *m/z*, (ROI 3, 3rd row) unknown at 105.94 *m/z*, and (ROI 4, 4th row) $[PbO_2H]^-$ at 240.97 *m/z*.

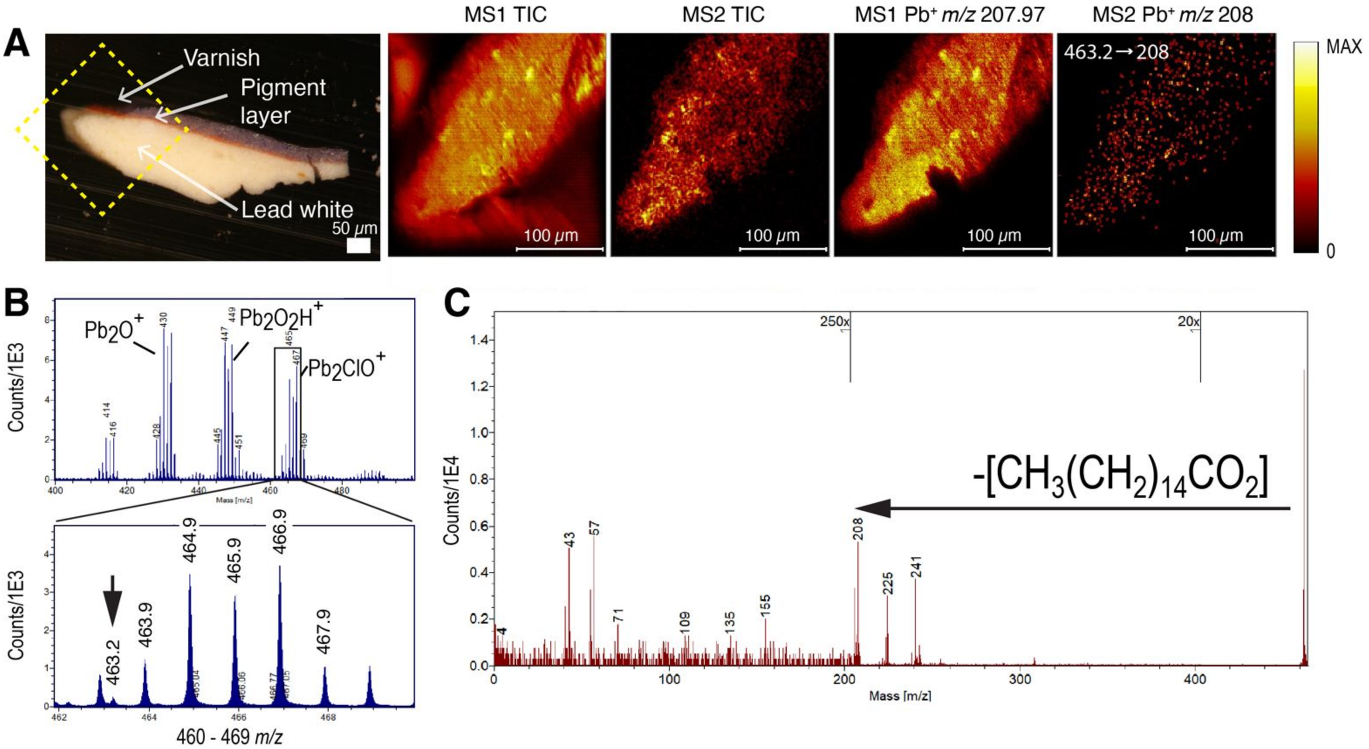

Figure S6. Parallel MS/MS imaging of palmitic acid lead soap from sample 3. A) Optical image of the sample and analysis area marked by a box. The ion images for MS1 (TIC and Pb+ at *m/z* 208.0) and MS2 (TIC and Pb+ at *m/z* 208.0) are presented. Scale bars at 100 µm. B) Mass spectra without tandem MS showing the palmitic acid lead soap peak at *m/z* 463.2. C) MS2 fragmentation of the palmitic acid lead soap peak at *m/z* 463.2 isolated using the tandem MS 1 Da window.
